# Multimodal prediction of trait emotional intelligence–Through affective changes measured using non-contact based physiological measures

**DOI:** 10.1371/journal.pone.0254335

**Published:** 2021-07-09

**Authors:** Vrinda Prajapati, Rajlakshmi Guha, Aurobinda Routray

**Affiliations:** 1 Advanced Technology Development Centre, Indian Institute of Technology Kharagpur, Kharagpur, West Bengal, India; 2 Centre for Education Technology, Indian Institute of Technology Kharagpur, Kharagpur, West Bengal, India; 3 Department of Electrical Engineering, Indian Institute of Technology Kharagpur, Kharagpur, West Bengal, India; Universita degli Studi di Pisa, ITALY

## Abstract

Inability to efficiently deal with emotionally laden situations, often leads to poor interpersonal interactions. This adversely affects the individual’s psychological functioning. A higher trait emotional intelligence (EI) is not only associated with psychological wellbeing, educational attainment, and job-related success, but also with willingness to seek professional and non-professional help for personal-emotional problems, depression and suicidal ideation. Thus, it is important to identify low (EI) individuals who are more prone to mental health problems than their high EI counterparts, and give them the appropriate EI training, which will aid in preventing the onset of various mood related disorders. Since people may be unaware of their level of EI/emotional skills or may tend to fake responses in self-report questionnaires in high stake situations, a system that assesses EI using physiological measures can prove affective. We present a multimodal method for detecting the level of trait Emotional intelligence using non-contact based autonomic sensors. To our knowledge, this is the first work to predict emotional intelligence level from physiological/autonomic (cardiac and respiratory) response patterns to emotions. Trait EI of 50 users was measured using Schutte Self Report Emotional Intelligence Test (SSEIT) along with their cardiovascular and respiratory data, which was recorded using FMCW radar sensor both at baseline and while viewing affective movie clips. We first examine relationships between users’ Trait EI scores and autonomic response and reactivity to the clips. Our analysis suggests a significant relationship between EI and autonomic response and reactivity. We finally attempt binary EI level detection using linear SVM. We also attempt to classify each sub factor of EI, namely–perception of emotion, managing own emotions, managing other’s emotions, and utilization of emotions. The proposed method achieves an EI classification accuracy of 84%, while accuracies ranging from 58 to 76% is achieved for recognition of the sub factors. This is the first step towards identifying EI of an individual purely through physiological responses. Limitation and future directions are discussed.

## Introduction

Emotions help in optimizing adaptation but are not always helpful and become dysfunctional when the wrong type of emotions are perceived and expressed at the wrong time or at the wrong intensity level, thus requiring efficient emotion regulation [1]. Inability to efficiently handle emotionally laden situations, leads to affect related problems in the long run. Mental health problems have their peak onset during young adulthood; they are prevalent in college students with the most common ones being substance abuse, anxiety, depression and other mood disorders. This may be because college corresponds to a challenging time as many students may face stressful experiences for the first time such as working, being in a significant relationship, and dealing with cultural differences [2–5]. Seventy five percent of those who will have a mental health disorder have had their first onset by the age of 25 years [6]. Regardless, there is inadequate treatment and lack of acknowledgment of mental health symptoms due to fear of personal stigma, denial (not perceiving treatment as urgent or essential), and lack of time, leading to persistence of these problems [7].

It has been found that people with higher levels of emotional intelligence are not only less prone to mental health problems but also typically achieve more positive life outcomes, such as psychological wellbeing, educational attainment, and job-related success. When faced with a stressful situation, emotionally intelligent individuals show a more adaptive response than those with low EI, such as reduced reactivity (less mood deterioration, less physiological arousal), and faster recovery once the threat has passed. EI is associated with better stress management, lower levels of psychological distress, higher resilience to peer pressure in connection to risky behaviour, less prone to substance abuse, and lower risk of mood disorders [8–10]. EI has also been found to reliably predict important outcomes like depression in adults [11]. In addition to better mental health, high EI is also associated with better physical health, career adaptability, decision making, job performance, job satisfaction, better quality of life [12, 13].

As Dr. Robert Tett reported “*EI can be assessed in a number of ways*. *A great way would be to have people face real or simulated situations that are emotionally charged and then just see how they react*. *E*.*g*. *Do they recognize their feelings*, *and can they describe them in words*? *Are their reactions appropriate in meeting the demands of a situation*, *or are they counterproductive*? *Do they react off-the-cuff*? *Do they show concern for others*? *How do they show that concern*? *And so forth*” [14]. Which is precisely the goal of this work, to predict EI level of an individual based on his/her physiological responses to emotionally charged situations.

## Rationale

Most importantly, higher EI has been found to be associated with willingness to seek professional and non-professional help for personal-emotional problems, depression and suicidal ideation [8]. Thus this makes it necessary to find out the low EI individuals and approach them to help them increase their EI and deal with emotions better. The problem of low treatment seeking in low EI college students may be addressed by the use of technology. Web based methods to screen college students for mood disorders have been found to increase the rate of help-seeking behaviour among at-risk students [2]. It has been found that although the self-report measures of EI have high validity and reliability, individuals tend to fake their responses in high stake situations, which varies as a function of degree of how important the consequences of test results could be expected to be, more high-stakes situations being associated with more faking [15]. If there is no subsequent consequence of responding to a psychological assessment questionnaire, the participant is likely to respond honestly, on the other hand, fake responses are likely if the consequence may be adverse, like losing a job or making a therapist appointment. Sometimes individuals may be unaware of their level of emotional skills due to which their responses to a self-report questionnaire may be biased and they will be less likely to identify and internalise feedback from others concerning their negative EI performance. This highlights the importance of developing systems that assesses, mood related and other problems through physiological measures, which are objective, unbiased and cannot be faked.

Significant research is being conducted on detecting anxiety [16], depression [17] and other mental health related issue through physiological measures [18, 19]. But in addition to that it would be beneficial to screen individuals for their emotional intelligence as well. Why should we wait till the onset of a mental health problem? Healthy students having low EI are more prone to mental health problems than their high EI counterparts, identifying low EI individuals and giving them the appropriate EI training will aid in preventing the onset itself [8–11].

## Emotions

Emotion is a psycho-physiological process triggered by conscious and or unconscious perception of an object or situation and is accompanied by various affective states giving rise to a set of physiological responses [20]. There is disagreement regarding how emotions are represented in the brain: either as discrete categories (happiness, sadness, disgust etc) or as points in a continuous dimensional space (valence and arousal). Although both the models posit that, processes mediated by the central nervous system give rise to the emotional states but their emergence is characterized via different mechanisms [21]. Even though people categorise their emotional experiences into discrete categories empirical evidence suggests that what exists in brain and body is affect which is continuous and emotions are constructed by multiple brain networks working together. Affect is defined as the underlying experience or sense of feeling, ranging from unpleasant to pleasant (valence), and from agitated to calm (arousal). Emotion is a much more complex mental construction [22]. As stated in the theory of constructed emotion “In every waking moment, your brain uses past experience, organized as concepts, to guide your actions and give your sensations meaning. When the concepts involved are emotion concepts, your brain constructs instances of emotion” [22]. Along with concepts, your interoceptive predictions provide information regarding the internal state of the body and produces basic affective feelings of pleasure, displeasure, arousal and calmness. The individual gives a name to these affective experiences based on the factors mentioned above [22–27].

A recent study showed that though results from neurological studies are supporting the notion that emotions are categorically organized response patterns, given the broad array of emotions considerable information beyond valence and arousal is required to differentiate emotional states [21]. We therefore used videos targeting discrete emotional states (joy, disgust, sadness, anger, and fear) for emotion elicitation, [21]. Since people experience an emotion when they conceptualize an instance of affective feeling, an emotional stimulus say, of fear will not produce the same sensations in everyone’s body, and will thus produce different experiences [23]. One of the factors that could lead to individual differences in affective experience is emotional intelligence [28].

### Measuring emotions

The most frequently used methods to assess emotional state of an individual are through self-report questionnaires and recording respondent’s physiological responses to stimuli which indicate activation of the autonomic nervous system. Despite significant research on the measurement characteristics of emotion self-reports and physiological measures, there is still doubt regarding the degree to which these 2 measures converge [29, 30]. Various researchers have found that self-report and physiological measures of emotion do not correlate implying that there may be important differences between the factors that drive responses to emotion self-reports and those that drive physiological reactions [31, 32]. Though physiological reactions occur prior to self-reports and possibly guide self-report, other factors like the tendency to engage in a socially desirable responding, ability to regulate their emotions, and lack of emotional self-awareness may influence the responses [29, 33]. In this study we also observe how EI affects emotional experience both physiologically and in self-report feedback form.

### Autonomic nervous system (ANS)

All aspects of emotion, whether it is generation, expression, experience, or recognition of emotion lead to ANS [34]. In addition to affective processes, ANS mediates patterns of brain activation in physiological, cognitive and behavioural processes as well [35]. The complex interplay between CNS and ANS and between the sympathetic and parasympathetic subsystems is modulated by descending, ascending, and bidirectional neural connections which can be described by a functional integrated model called the Central Autonomic Network (CAN). Through this model/system, the brain directly/indirectly controls visceromotor, neuroendocrine, and behavioural responses that are critical for goal-directed behaviour, adaptability, and health and also regulates for homeostasis, facilitates short-term deviations from homeostasis by allocating resources to cope with new tasks, and coordinates the continuous bidirectional flow of information [35]. The primary output of CAN is mediated by sympathetic and parasympathetic neurons which at the sino atrial node produce the complex beat to beat variability that characterises the heart rate time series. HRV is directly related to the output from CAN [36].

ANS is an extremely dynamic system, and the major organ systems innervated by it are–cardiovascular, respiratory and electro dermal systems, and changes in their functioning reflect activation levels of SNS (sympathetic nervous system) and PNS (Parasympathetic nervous system). An event occurrence typically causes a change in the cyclical functioning of the cardiovascular and respiratory systems; also the responses of these 2 systems can be measured using non-contact based sensors, thus in this work we will measure the psychophysiological response patterns to emotion using cardiovascular and respiratory parameters [37].

#### Cardiovascular activity

The ease with which an individual can transition between various arousal states is dependent on the ability of the ANS to rapidly vary heart rate (HR) which is reflected as heart rate variability (HRV) and acts as an indicator for autonomic flexibility and emotion regulation (ER) capacity [38]. HRV at rest indicates the ER capacity of an individual which is a major component of EI and, a lower resting state HRV is associated with greater difficulties with emotion regulation [39, 40]. HRV is the beat to beat variability and can be calculated using both time domain and frequency domain measures. Most commonly used time domain measures for short term recordings are RMSSD and SDNN which indicate high frequency variations/ vagal modulation and overall variability respectively. In the study of emotions and autonomic responses special attention has been given to heart rate variability due to tonic parasympathetic influence emanating from the Vagal nerve as it is responsive to environmental demands and underlies the ability to regulate emotions and respond adaptively to emotional provocation [41].

#### Respiratory activity

Respiratory patterns vary with valence and arousal of emotion [42] and reflect the dimension of emotional response that is linked to response requirements of the emotional situations. The breathing cycle can be characterized by tidal volume (Vt) (i.e. the volume that is displaced during one breath), duration of inspiration (Ti), duration of expiration (Te), and total cycle duration (Ttot), and is regulated mainly by a central inspiratory drive mechanism, which determines the intensity of the inspiratory stimulus, and a periodic rhythm generator which cyclically switches the drive mechanism on and off. An increase in inspiratory drive primarily increases Vt/Ti (the mean inspiratory flow rate), respiratory minute volume (Vmv). Although rhythm generator is a main determinant of duration of inspiratory phase, but if Vt increases greatly rhythm generator is overruled and termination of inspiration is brought about by an afferent vagal reflex, that is triggered by the stretch receptors in the lungs. Expiration duration is primarily a function of the degree of filling of the lungs during the previous inspiration, and of the resistance that the outflow of air encounters, and thus may be regarded as a passive process [43].

### Emotional intelligence

Emotional intelligence (EI) is the ability, capacity, or skill to identify, assess, and manage the emotions of one’s self, of others and of groups [44]. It’s the juncture at which cognition and emotion meet, and facilitates our capacity for resilience, motivation, empathy, reasoning, stress management, communication, and our ability to read and navigate a plethora of social situations and conflicts [45]. Emotional intelligence refers adaptive emotional functioning. Meta analytic studies have found that higher emotional intelligence is associated with a variety of better outcomes, including employment and academic performance, and better mental and physical health [46].

#### History of EI

EI emerged as a major psychological construct in 1990 when Salovey and Mayer defined it as “the ability to monitor one’s own and others’ feelings and emotions, to discriminate among them and to use this information to guide one’s thinking and actions” [47]. It gained popularity when Daniel Goleman published the book ―Emotional Intelligence: Why It can Matter More than IQ [48–50].

Later, Mayer and Salovey considered EI as a form of pure intelligence and argued that it is best conceived as ability and assessed through performance tests that assess maximum performance [46]. But realising the drawbacks associated with the objective nature of questions in performance based/ability EI test, various researchers started utilizing self-report questions that assessed behavioural tendencies [51, 52]. Other measures of EI were developed that started incorporating additional factors that would fall under a broader definition of EI along with the typical EI facets [47]. Reuven Bar-On conceptualised EI as an array of non-cognitive capabilities, competencies, and skills that influence one’s ability to succeed in coping with environmental demands and pressures [48–50, 53] Petrides and Furnham posited that emotional intelligence can be conceptualized as a trait or as typical functioning, situated between the upper and lower extremes of personality hierarchies.

#### Types of EI

EI types can be differentiated on the basis of model or measurement technique. The ability, trait and mixed models of EI differ in what elements they consider constitute EI. When considering measurement technique EI can be divided into ability and trait EI, which differ in method used to measure the construct (questionnaire or performance based) and not on the facets that the various models encompass. In short, distinction between EI measures is unrelated to the distinction between EI models [54]. While ability EI is objective and performance based, a trait approach to assessing emotional intelligence draws on self or other’s reports to gather information regarding the display of emotional intelligence characteristics in daily life [55, 56]. *Ability EI* is assessed via performance tests and captures what an individual is capable of doing in an emotional situation measured via maximum performance tests. The problem with this approach is that it uses scoring procedures like “consensus” and “expert scoring” to create correct responses among a number of equally logical alternatives leading to various problems repeatedly noted in literature. It is unclear whether the scores reflect confounding with vocabulary size, conformity to social norms, theoretical knowledge about emotions, stereotypical judgements or some other factors [57].

*Trait EI* focuses on emotion related dispositions and affect related aspects of personality and captures how much of this knowledge/competence translates into practice [58]. Trait EI theory recognises the inherent subjectivity of emotional experience and maintains that certain profiles will be advantageous in some contexts but not in others [57]. Trait EI is currently measured only using self-report questionnaires or via interview with psychologists.

#### Measures of EI

According to the original four-branch model of emotional intelligence [59] by Salovey and Mayer, EI consists of the interrelated functions of (a) accurately perceiving emotion in the self and others; (b) using emotion to assist thinking, including decision making; (c) understanding emotion in the self and others; and (d) effectively managing emotion in the self and others. Researchers have pointed out that both ability and trait conceptualization share value and can be complimentary [46, 60] Moreover both these concepts can fit within this four-branch model of emotional intelligence and are important dimensions of adaptive emotional functioning [55]. An individual may have, for example, the ability to assist others regulate emotions (measured through ability measures), but not typically use this ability (measured through trait measures) for motivational or other reasons.

Some of the widely used trait EI measures include TEIque, SSEIT, EQi. Most of these measures based on the different models of EI incorporate additional emotion related facets, competencies and create combinations of personality traits that are particularly effective in situations with emotional and social implications [61]. But fundamentally digress from the core concept of EI, staying in the personality domain and the term ‘trait’ in trait EI became associated with personality and not just as a measurement technique difference [62, 63].

#### Physiological correlates of EI

Various affective studies have attempted to investigate the association between Emotional intelligence and physiological emotional response. High trait EI individuals have been found to use adaptive strategies to down regulate negative emotions like stress, anger, sadness, fear, jealousy and shame; and to maintain positive ones like joy [58]. Emotionally Intelligent individuals use adaptive attention processing patterns and have higher emotional attention regulation, as they pay more attention to positive emotional stimuli relative to negative and neutral stimuli [64, 65]. They have reduced HPA (hypothalamic–pituitary–adrenal) axis reactivity, which buffers the effect of stress by lowering the increase in heart rate in response to stress [66, 67], and greater grey matter volume and density in ventromedial prefrontal cortex (vmPFC) which is responsible for emotional processing [68] With respect to functional correlates, trait EI is associated with neurological activity in both resting and active/emotionally laden conditions with higher trait EI individuals showing greater resting left frontal activation [69].

## Our work

This work builds on and examines the influence of level of EI (measured using SSEIT) on emotional autonomic response patterns, namely cardiovascular and respiratory measures, and can be divided into 2 parts. We first conduct an experiment to understand the relation between autonomic (cardiac and respiratory) responses and reactivity to emotion elicitation with the level of EI using cardiovascular and respiratory measures. The reactivity is measured as the change in the autonomic parameters from baseline to emotion elicitation. An increase and decrease in these parameters is referred to as activation and withdrawal respectively. Secondly, we build a model to predict EI level of an individual using these measures. More specifically the data collected during first study is used to classify user’s EI level (high or low), with the time domain cardiac and respiratory parameters indicating response, and the difference in these parameters from baseline to emotional state indicating reactivity, observed while viewing film clips of 5 basic emotions (joy, disgust, sadness, anger, and fear). We designed a study with movie scenes as they have been found to be one of the most effective method of mood induction as they can generate a dynamic context using stimuli that are similar to those in real life, and without the ethical problem that arise when manipulating emotions, [70] can elicit discrete emotions and is also very effective in prolonged maintenance of both subjective and physiological changes in emotion [71, 72]. Also the data is recorded using commercial sensors to ensure ecological validity and scalability of the employed framework for large scale profiling applications. To our knowledge, this is the first work to connect EI and emotional physiological responses using a machine learning approach. Screening individuals for their level of emotional intelligence, will not only aid in identifying low EI individuals with depression anxiety etc. as all these constructs are related, but will also help in finding individuals who are at risk of developing mood disorders due to low EI (which hinders their ability to handle everyday emotional situations) and provide them with appropriate training and guidance.

## Materials and methods

### Participants

Fifty one college students (M_age_ = 25.15, SD _age_ = 2.97, 22 females) participated in the study. Exclusion criteria included any ongoing physical or mental health problems and or use of medications, alcohol or caffeine (on the day of the experiment). Healthy participants who had proper sleep the previous night, had timely breakfast and lunch, were not experiencing any physical or mental discomfort such as headache, nausea, cough and cold, low mood or pain in any part of the body were included in the study. The subject sample consisted of young adults in the age range 18 to 30 and with near equal number of males and females. The experiment was conducted at the same time every day between 4:00 pm to 6:00 pm. The sample size of 51 was decided on the basis of power analysis by G*Power software with α: 0.05 and β: 0.80 [73]. All the participants signed an informed consent form before taking part in the study, and were also informed that they had the right to quit the experiment at any time. They were debriefed about the purpose of the study at the end of the experiment. The experimental procedure and the video content shown to the participants were approved by the Institutional Ethics Committee (IEC) of IIT Kharagpur.

### Measures

#### Physiological measures–FMCW radar sensor

Electromagnetic radars have shown potential to be used for remote sensing of bio signals such as breathing and heartbeat signals in a more easy and comfortable way than contact based and wearable devices [74, 75]. Currently 3 main types of radar systems for vital signs monitoring are available—pulse radar (ultra-wide band radar), continuous wave (CW) Doppler radar, and frequency modulated continuous wave (FMCW) radar [75]. Compared to CW and pulse radars, the FMCW radar has a higher range and speed resolution, and its ability to distinguish multiple targets and extract target fretting information, makes it a mainstream choice in the field of life detection, [75] also mm-wave frequencies give high-resolution sensing of displacements in an environment in the order of sub-mm changes [76]. Therefore this paper uses Texas Instruments (TI) mm-wave FMCW radars which have shown to measure respiration and heart signals with high accuracy which is comparable to traditional contact based sensors (93% when compared to contact based sensors like airflow sensor, and smart bracelet Mi 3; and >80% when compared to ECG) [74–76]. The physiological data was recorded using the *FMCW radar sensor*, mmWave TI sensor (AWR1642). AWR1642 Boost is a single chip automotive radar sensor with built in micro controller using FMCW technology. It is a high frequency module working in 76 GHz to 81 GHz band. This module has two transmitter and four receiver antennas for the data collection. It is a real-time millimetre-wave radar-based, non-contact vital sign monitoring system that is capable of detecting the heart and breath waveform and has a sampling rate of 20 frames per second. It provides high accuracy in the range of 0.6 to 1 m [77]. It has a built in DSP and ARM processor for post processing and gives accurate heartbeat waveforms and respiratory waveforms. During experiment the sensor was kept inside a black box.

**Fig 1:**
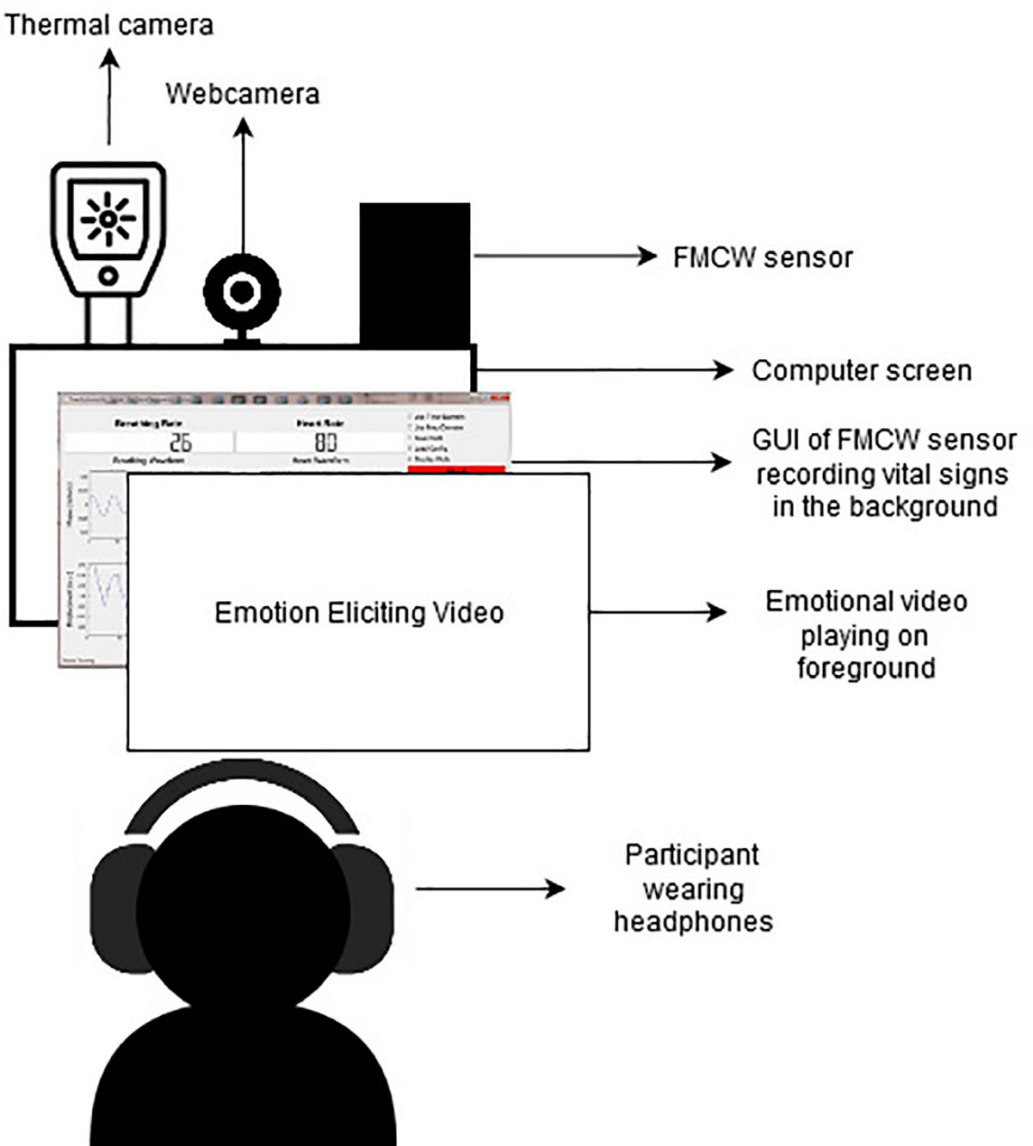
Experiment setup.

The FMCW radar sensor includes the radio frequency (RF) transmit (TX) and receive (RX), analog clocking, and digital circuit—analog-to-digital converters (ADCs), microcontrollers (MCUs), and digital signal processors (DSPs). It sends a linear FMCW signal generated by the synthesizer. The radar signal gets reflected when it encounters an object. Then, the orthogonal receiver is responsible for capturing the echo signal and orthogonally mixing it with the transmitted signal. A low-pass filter is used to filter out the high frequency part and obtain the IF signal. Finally, the IF signal is sampled by an ADC [75]. The heart waveform is later analysed and parameters such as heart rate (HR) and heart rate variability (RMSSD) were extracted by the standardised methods [78] using MATLAB. Similarly respiratory waveform was analysed to obtain parameters such as inspiration time (Ti), expiration time (Te), total breath time (Ttot), and tidal volume (Vt). Thermal facial videos were also recorded, but that data has not been included in the current study. A diagram of the experimental setup showing where the sensors were placed is shown in Fig 1 below.

#### Psychological measures–SSEIT and Feedback form

*1*. *SSEIT (Schutte Self report Emotional Intelligence Test)*. Trait EI was measured using the *Schutte Self Report Emotional Intelligence Test (SSEIT)*. It is a self-report TEI scale which consists of 4 factors—perception of emotion, managing own emotions, managing other’s emotions, and utilization of emotion. It is a 33-item self-report inventory in which the respondents rate themselves using a 5-point scale. This scale was chosen as it has been found to be distinct from standard personality factors [28].

The 4 subscales for the 4 factor structure of this scale is the most widely used structure and is based on the factor analtic studies by Petrides and Furnham [79], Ciarrorchi [28], and Saklofske et al. Some studies argued against the internal consistency of ‘utilization od emotion’ subscale, suggesting the use of a 3 factor model instead [55]. But a recent study revealed evidence for the usefulness of SSEIT and its 4 subscales for measuring EI and its subfcators respectively [59]. Psychometric properties of the scale are given below:

- Internal consistency: Various studies found the internal consisitency of the scale to be .87. Based on the responses from university students, the internal consisitency for the subscales was found to be Perception of Emotion, .80; Managing Own Emotions, .78; Managing Others’ Emotions, .66; and Utilisation of Emotion, .55 (the alpha for this sub scale was not reported) [55].
- Test retest reliability: A two-week test–retest reliability of .78 was found for the total scale scores [55].
- Validity: Significant convergent and divergent validity has been found for this scale through multiple studies [55].

*2*. *Feedback form*. *Emotional Experience Feedback form* was given at the end of each emotion eliciting video, in which the respondent had to rate how emotional he/she felt while watching the video (intensity) and also the valence and arousal they experienced. The ratings ranged from 1 to 5, with 1 indicating not at all and 5 indicating extremely.

#### Rationale behind choosing SSEIT

While aiming to develop a physiological measure of EI we decided to go with a measure based on the original framework of EI [62] which covers all the important facets of EI and at the same time captures the subjective nature of emotions. SSEIT attempts to assess characteristic, or trait, emotional intelligence and is based on Salovey and Mayer’s (1990)original model [54, 55] Although it was designed to measure overall EI, it performs better as a multidimensional scale measuring 4 distinct factors including optimism/mood regulation (regulation of emotions in self), appraisal of emotions (perception of emotion), social skills (regulating emotions in others) and utilization of emotions (strategically utilizing emotions) which has been confirmed [51, 80].

The scale may be used to help individuals who are at risk for performing poorly at tasks that require emotional intelligence, (as in establishing themselves in a new setting such as a college). The finding that emotional intelligence scale scores predicted first-year college grades suggests the ability of the scale to help identify these individuals. Once identified, these at-risk individuals might benefit from special guidance, training or support [52]. The scale has also been found to be distinct from standard personality factors [28].

Moreover, the explanatory models of personality that view surface dimension of personality have a distal basis in emotion control. Meaning, that an individual’s personality is based on how he/ she handles emotions [81] Thus we decided to use a measure of EI which stems from the original EI model and not from the personality model. Also despite the correlations found with some personality factors, the SSEIT has been found to be distinct from personality [10].

### Procedure

**Fig 2:**
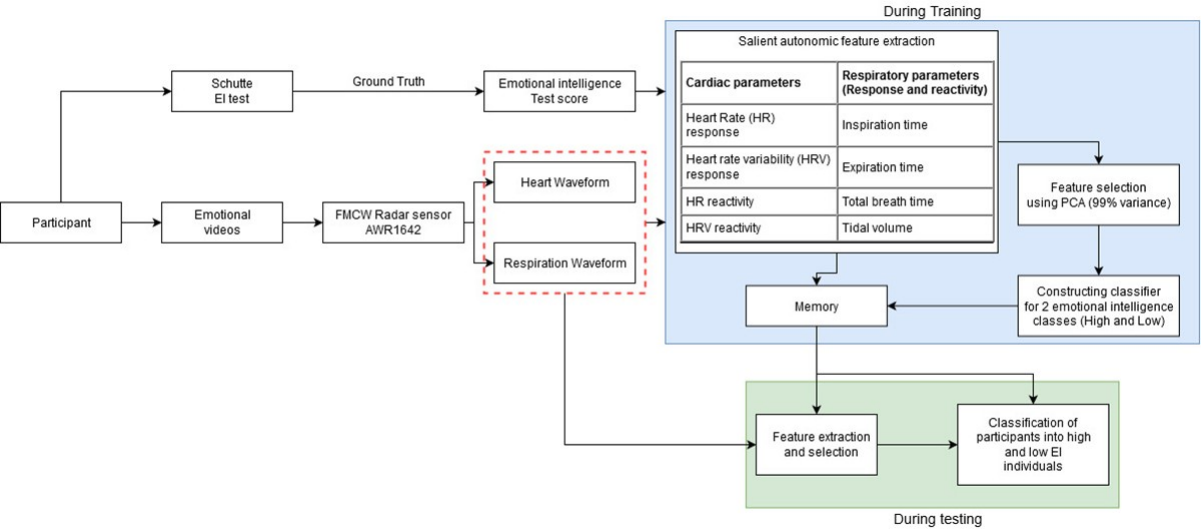
Proposed method for EI prediction.

An overview of our EI prediction framework is presented in Fig 2 below. To study the EI-Affect relationship we performed a study where user’s physiological responses were recorded first at baseline and then as they viewed 5 affective film clips chosen from standardized databases. Explicit feedback in the form of intensity, valence and arousal of emotion experienced was obtained after they viewed each movie clip. User’s EI measures were compiled using Schutte Self Report Emotional Intelligence Test (SSEIT).

One Monitor with webcam and a FMCW radar sensor was used for the experiment. Thermal camera was also used to record facial thermal videos of the participant’s but that data has not been included in this paper. Following an informed consent form, and SSEIT questionnaire, the user was asked to sit in front of the monitor, wear headphones and click a start button on the screen which simultaneously started a video clip (neutral video followed by the film clip), the webcam and the FMCW sensor.

Each user performed the experiment in 5 sessions, one session for each emotion lasting about 5 minutes occurring on separate days. Each emotional film clip was preceded by a 1 minute neutral video of cattle grazing on a farm to bring the participants to a baseline state. The physiological recordings done during this period served as baseline. The physiological parameters taken while watching the emotional videos served as the emotional response and the difference in parameters from baseline to emotional response served as emotional reactivity. On viewing each clip users provided their feedback affective ratings.

**Fig 3:**
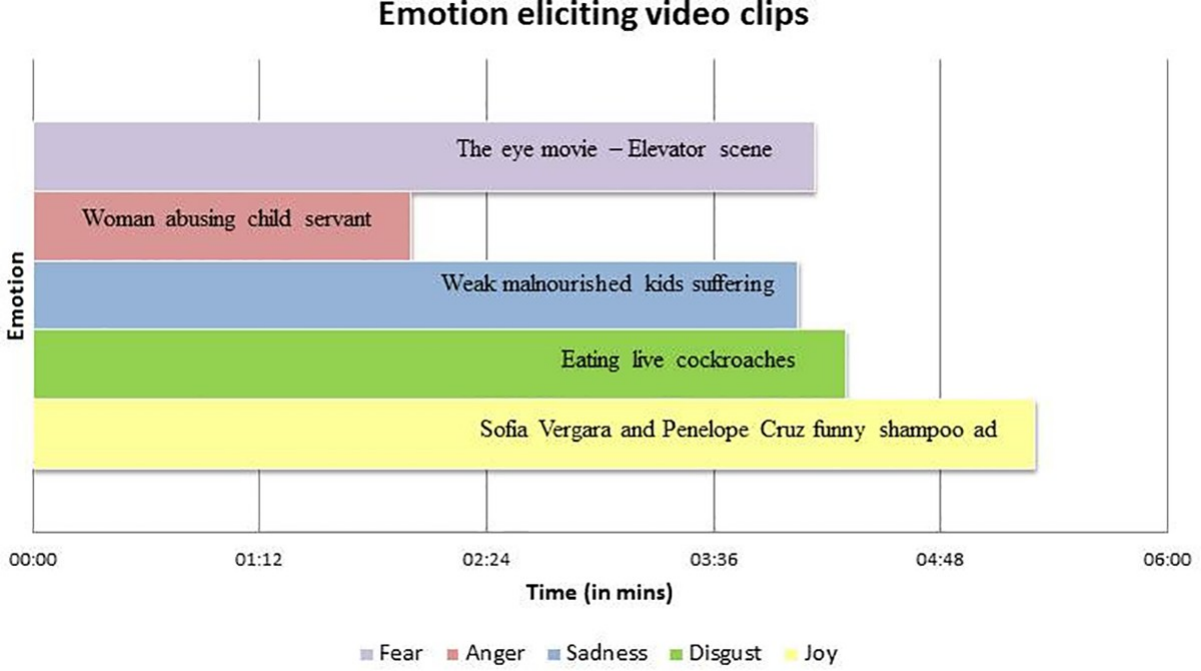
Emotion eliciting video clips.

Stimuli: We adopted 5 movie clips from earlier studies (such as The Emotional Movie Database [82], Emotion Elicitation Using Films [83], and The Indian Spontaneous Expression Database for Emotion Recognition [84]) for our study. These clips are between 1–5 minutes long and have shown to efficiently induce the intended emotions in the user. Some videos were found to elicit multiple emotions, which were rejected as they may lead to a mixed emotional expression. The final clips used are mentioned in Fig 3.

### Physiological feature extraction

The heart beat waveform obtained was filtered using the Savitzky-Golay filter for noise and random body movement’s artefacts reduction and the SNR enhancement without distorting the heartbeat signal. R peaks were detected to obtain the RR intervals from which time domain features namely RMSSD (Root mean square of successive differences), and HR (Heart rate) were extracted for the overall trial (cardiac autonomic response) and the difference in RMSSD and HR from baseline to emotional state (cardiac autonomic reactivity) was also calculated. HRV at baseline was also taken as it is an objective indicator of emotion regulation capacity [40]. Respiratory waveform was first smoothed, followed by peak and valley detection for inspiration and expiration respectively. Four parameters were then calculated–Inspiration time (time from each expiration valley to the next inspiration peak), Expiration Time (Time from each Inspiration peak to the next expiration valley), Tidal Volume (height of waveform for each breathing cycle), and Total breath time (time of each breath cycle). Similar to cardiac reactivity, respiratory reactivity was also measured as difference in the four parameters from baseline to emotional state. Both cardiac and respiratory reactivity are represented with Δ. In total 6 features were obtained for baseline and for each emotion elicitation.

### Statistical analysis

All the data was screened for potential outliers and missing values. There was no missing data or outliers. Inspection of skew, kurtosis, and histograms for the variables indicated the presence of reasonably normal distributions among variables. One sampled t–test was conducted on the feedback form ratings to see if they were significantly different from 0 for all the emotion elicitations. One way ANOVA was used on all the variables (EI scores, physiological response and reactivity and emotion feedback scores) to find out if there were any significant gender differences. In order to study the association between EI scores and autonomic (cardiac and respiratory) response and reactivity, bivariate correlations were performed between SSEIT scores and autonomic responses at baseline, and autonomic response and reactivity to the 5 emotions. All the statistical analysis was performed using SPSS software.

### SVM classifier

Machine learning systems are systems that learn from known data, called training phase, and return a model that can be used to map/classify unknown input data into a category [85]. Various classifiers like Decision trees, K-nearest neighbours, SVM use different decision algorithms for these classification tasks, we chose to use SVM, as they have proven to be highly effective in various studies of affective computing, and efficiently maintain sufficient flexibility with regard to their internal main parameter optimization [86, 87].

A support vector machine classifies binary classes by finding and using a class boundary, the hyper plane, maximizing the margin in the given training data. The training data samples along the hyper planes near the class boundary are called support vectors, and the margin is the distance between the support vectors and the class boundary hyperplanes. The SVM are based on the concept of decision planes (that separates between assets of objects having different class memberships) that define decision boundaries. SVM makes the decision boundary in such a way that the separation between the 2 classes is as wide as possible.

## Results

Since the data was assuming normality we used parametric tests for our statistical analysis. Subjects were asked to rate the extent to which they felt the intended emotion on a 5 point scale (1: not at all, 5: extremely) immediately after the video got over. Feedback form ratings indicated that the intended emotion was successfully induced with varying intensity. One sampled t–test showed that the ratings were significantly different from 0 for all the emotion elicitations, Table 1 below present’s descriptive statistics for the EI scores and feedback form ratings.

**Table 1:** Descriptive statistics for SSEIT scores and feedback form rating.

Variable	Mean	Std. Deviation
**Gender**	0.44	0.50
**Perception of emotion**	38.14	5.7
**Managing own emotions**	36.86	4.75
**Managing other’s emotions**	32.18	3.68
**Utilization of emotion**	24.56	3.47
**Total EI**	131.74	13.61
**Feedback Joy**	3.55	1.07
**Feedback Disgust**	3.62	1.38
**Feedback Sad**	3.48	1.19
**Feedback Anger**	3.86	1.16
**Feedback Fear**	3.39	1.14

### Gender differences

One way ANOVA was used to observe any significant gender differences regarding (Table 2) EI scores, physiological response and reactivity at (Table 3) baseline, (Table 4) joy, (Table 5) disgust, (Table 6) sadness, (Table 7) anger, (Table 8) fear, and (Table 9) emotion feedback scores.

**Table 2:** ANOVA for SSEIT scores of males and females.

Parameter	Male mean (sd)	Female mean (sd)	Source	Df	F	Sig.
**Total EI**	131.96 (15.887)	131.45 (10.396)	**Between groups**	1	.017	.897
			**Within groups**	48		
			**Total**	49		
**Perception of emotion**	38.93 (6.744)	37.14 (4.257)	**Between groups**	1	1.181	.283
			**Within groups**	48		
			**Total**	49		
**Managing own emotions**	36.75 (5.104)	37 (4.386)	**Between groups**	1	.033	.856
			**Within groups**	48		
			**Total**	49		
**Managing other’s emotions**	32.14 (4.187)	32.23 (3.023)	**Between groups**	1	.006	.937
			**Within groups**	48		
			**Total**	49		
**Utilization of emotion**	24.14 (3.988)	25.09 (2.671)	**Between groups**	1	.918	.343
			**Within groups**	48		
			**Total**	49		

EI: Emotional intelligence; sd: standard deviation; Df: degree of freedom; Sig: significance.

**Table 3:** ANOVA for baseline cardiac and respiratory responses of males and females.

Parameter	Male mean (sd)	Female mean (sd)	Source	Df	F	Sig.
**Baseline HR**	76 (12.829)	76.95 (12.975)	**Between groups**	1	.062	.804
			**Within groups**	44		
			**Total**	45		
**Baseline RMSSD**	56 (13.497)	59.9 (11.054)	**Between groups**	1	1.123	.295
			**Within groups**	44		
			**Total**	45		
**Baseline Ti**	1.35 (0.714)	1.14 (0.359)	**Between groups**	1	1.405	.243
			**Within groups**	42		
			**Total**	43		
**Baseline Te**	1.57 (0.788)	1.43 (0.507)	**Between groups**	1	.458	.502
			**Within groups**	42		
			**Total**	43		
**Baseline Vt**	0.57 (0.507)	0.38 (0.498)	**Between groups**	1	1.476	.231
			**Within groups**	42		
			**Total**	43		
**Baseline Ttot**	3 (1.243)	2.76 (0.539)	**Between groups**	1	.657	.422
			**Within groups**	42		
			**Total**	43		

sd: standard deviation; Df: degree of freedom; Sig: significance; HR: Heart rate; RMSSD: Root mean square of successive differences; Ti: Inspiration time; Te: Expiration time; Vt: Tidal volume; Ttot: Total breath time.

**Table 4:** ANOVA for autonomic (cardiac and respiratory) response and reactivity during joy of males and females.

Parameter	Male mean (sd)	Female mean (sd)	Source	Df	F	Sig.
**Joy HR**	82.86 (3.577)	81.19 (7.118)	**Between groups**	1	1.153	.288
			**Within groups**	47		
			**Total**	48		
**Joy RMSSD**	58.59 (8.219)	53.38 (7.902)	**Between groups**	1	4.911	.032*
			**Within groups**	46		
			**Total**	47		
**Joy Ti**	1.54 (1.036)	1.33 (0.483)	**Between groups**	1	.687	.411
			**Within groups**	47		
			**Total**	48		
**Joy Te**	1.82 (1.416)	1.57 (0.507)	**Between groups**	1	.595	.444
			**Within groups**	47		
			**Total**	48		
**Joy Vt**	0.75 (0.441)	0.62 (0.498)	**Between groups**	1	.948	.335
			**Within groups**	47		
			**Total**	48		
**Joy Ttot**	3.61 (1.75)	2.86 (0.793)	**Between groups**	1	3.331	.074
			**Within groups**	47		
			**Total**	48		
**Δ Joy HR**	6.64 (13.391)	4.3 (14.79)	**Between groups**	1	.309	.581
			**Within groups**	43		
			**Total**	44		
**Δ Joy RMSSD**	1.63 (13.021)	-8.25 (12.937)	**Between groups**	1	6.311	.016*
			**Within groups**	42		
			**Total**	43		
**Δ Joy Ti**	0.17 (1.23)	0.25 (0.444)	**Between groups**	1	.069	.795
			**Within groups**	41		
			**Total**	42		
**Δ Joy Te**	0.48 (1.039)	0.05 (0.51)	**Between groups**	1	2.804	.102
			**Within groups**	41		
			**Total**	42		
**Δ Joy Vt**	-0.09 (0.288)	0.01 (0.324)	**Between groups**	1	.867	.357
			**Within groups**	41		
			**Total**	42		
**Δ Joy Ttot**	0.78 (1.594)	0.30 (0.801)	**Between groups**	1	1.500	.228
			**Within groups**	41		
			**Total**	42		

sd: standard deviation; Df: degree of freedom; Sig: significance; HR: Heart rate; RMSSD: Root mean square of successive differences; Ti: Inspiration time; Te: Expiration time; Vt: Tidal volume; Ttot: Total breath time; Δ: Reactivity parameters.

**Table 5:** ANOVA for autonomic (cardiac and respiratory) response and reactivity during disgust of males and females.

Parameter	Male mean (sd)	Female mean (sd)	Source	Df	F	Sig.
**Disgust HR**	82.81 (3.611)	82 (3.044)	**Between groups**	1	.646	.426
			**Within groups**	44		
			**Total**	45		
**Disgust RMSSD**	60.88 (9.454)	60.85 (8.028)	**Between groups**	1	.000	.990
			**Within groups**	44		
			**Total**	45		
**Disgust Ti**	1.2 (0.5)	1,65 (1.182)	**Between groups**	1	2.972	.092
			**Within groups**	43		
			**Total**	44		
**Disgust Te**	1.72 (0.614)	1.65 (0.671)	**Between groups**	1	.133	.717
			**Within groups**	43		
			**Total**	44		
**Disgust Vt**	0.6 (0.5)	0.6 (0.503)	**Between groups**	1	.000	1.000
			**Within groups**	43		
			**Total**	44		
**Disgust Ttot**	3.24 (0.926)	3.5 (1.539)	**Between groups**	1	.493	.487
			**Within groups**	43		
			**Total**	44		
**Δ Disgust HR**	6.83 (13.454)	5.63 (13.541)	**Between groups**	1	.084	.773
			**Within groups**	41		
			**Total**	42		
**Δ Disgust RMSSD**	3.88 (16.546)	-1.79 (13.419)	**Between groups**	1	1.463	.233
			**Within groups**	41		
			**Total**	42		
**Δ Disgust Ti**	0.1 (0.926)	0.53 (1.264)	**Between groups**	1	2.357	.133
			**Within groups**	39		
			**Total**	40		
**Δ Disgust Te**	0.18 (1.097)	0.32 (0.946)	**Between groups**	1	.172	.680
			**Within groups**	39		
			**Total**	40		
**Δ Disgust Vt**	0.05 (0.375)	-0.11 (0.459)	**Between groups**	1	1.339	.254
			**Within groups**	39		
			**Total**	40		
**Δ Disgust Ttot**	0.27 (1.486)	1 (1.732)	**Between groups**	1	2.095	.156
			**Within groups**	39		
			**Total**	40		

sd: standard deviation; Df: degree of freedom; Sig: significance; HR: Heart rate; RMSSD: Root mean square of successive differences; Ti: Inspiration time; Te: Expiration time; Vt: Tidal volume; Ttot: Total breath time; Δ: Reactivity parameters.

**Table 6:** ANOVA for autonomic (cardiac and respiratory) response and reactivity during sadness of males and females.

Parameter	Male mean (sd)	Female mean (sd)	Source	Df	F	Sig.
**Sad HR**	81.2 (4.193)	81.33 (3.152)	**Between groups**	1	.014	.905
			**Within groups**	44		
			**Total**	45		
**Sad RMSSD**	55.12 (8.96)	53.57 (8.459)	**Between groups**	1	.359	.552
			**Within groups**	44		
			**Total**	45		
**Sad Ti**	1.56 (0.583)	1.71 (0.717)	**Between groups**	1	.648	.425
			**Within groups**	44		
			**Total**	45		
**Sad Te**	2.36 (1.15)	1.81 (0.602)	**Between groups**	1	3.902	.055
			**Within groups**	44		
			**Total**	45		
**Sad Vt**	0.56 (0.712)	0.33 (0.483)	**Between groups**	1	1.533	.222
			**Within groups**	44		
			**Total**	45		
**Sad Ttot**	3.72 (1.514)	3.62 (1.071)	**Between groups**	1	.066	.799
			**Within groups**	44		
			**Total**	45		
**Δ Sad HR**	5.2 (13.288)	4.19 (13.984)	**Between groups**	1	.063	.803
			**Within groups**	44		
			**Total**	45		
**Δ Sad RMSSD**	-0.96 (13.776)	-6.43 (13.422)	**Between groups**	1	1.841	.182
			**Within groups**	44		
			**Total**	45		
**Δ Sad Ti**	0.26 (0.689)	0.48 (0.602)	**Between groups**	1	1.210	.278
			**Within groups**	42		
			**Total**	43		
**Δ Sad Te**	0.78 (1.22)	0.39 (0.66)	**Between groups**	1	1.683	.202
			**Within groups**	42		
			**Total**	43		
**Δ Sad Vt**	0.05 (0.57)	-0.02 (0.17)	**Between groups**	1	.280	.600
			**Within groups**	42		
			**Total**	43		
**Δ Sad Ttot**	0.82 (1.63)	0.93 (1.01)	**Between groups**	1	.074	.788
			**Within groups**	42		
			**Total**	43		

sd: standard deviation; Df: degree of freedom; Sig: significance; diff: difference; HR: Heart rate; RMSSD: Root mean square of successive differences; Ti: Inspiration time; Te: Expiration time; Vt: Tidal volume; Ttot: Total breath time; Δ: Reactivity parameters.

**Table 7:** ANOVA for autonomic (cardiac and respiratory) response and reactivity during anger of males and females.

Parameter	Male mean (sd)	Female mean (sd)	Source	Df	F	Sig.
**Anger HR**	81.96 (4.158)	81.24 (4.06)	**Between groups**	1	.351	.556
			**Within groups**	44		
			**Total**	45		
**Anger RMSSD**	55.2 (10.544)	47.1 (9.165)	**Between groups**	1	7.587	.009**
			**Within groups**	44		
			**Total**	45		
**Anger Ti**	1.21 (0.415)	1.5 (0.827)	**Between groups**	1	2.298	.137
			**Within groups**	42		
			**Total**	43		
**Anger Te**	1.63 (1.056)	1.7 (0.47)	**Between groups**	1	.086	.770
			**Within groups**	42		
			**Total**	43		
**Anger Vt**	0.5 (0.511)	0.4 (0.503)	**Between groups**	1	.424	.518
			**Within groups**	42		
			**Total**	43		
**Anger Ttot**	2.96 (0.955)	3.1 (0.718)	**Between groups**	1	.299	.587
			**Within groups**	42		
			**Total**	43		
**Δ Anger HR**	7 (14.529)	4.14 (14.378)	**Between groups**	1	.429	.516
			**Within groups**	42		
			**Total**	43		
**Δ Anger RMSSD**	1.22 (17.368)	-12.76 (13.561)	**Between groups**	1	8.736	.005**
			**Within groups**	42		
			**Total**	43		
**Δ Anger Ti**	-0.122 (0.577)	0.338 (0.724)	**Between groups**	1	5.107	.029*
			**Within groups**	39		
			**Total**	40		
**Δ Anger Te**	0.319 (1.19)	0.21 (0.408)	**Between groups**	1	.148	.702
			**Within groups**	39		
			**Total**	40		
**Δ Anger Vt**	-0.017 (0.29)	-0.05 (0.26)	**Between groups**	1	.212	.648
			**Within groups**	39		
			**Total**	40		
**Δ Anger Ttot**	0.12 (0.826)	0.56 (0.851)	**Between groups**	1	2.810	.102
			**Within groups**	39		
			**Total**	40		

sd: standard deviation; Df: degree of freedom; Sig: significance; HR: Heart rate; RMSSD: Root mean square of successive differences; Ti: Inspiration time; Te: Expiration time; Vt: Tidal volume; Ttot: Total breath time; Δ: Reactivity parameters.

*p<0.05

**p<0.01.

**Table 8:** ANOVA for autonomic (cardiac and respiratory) response and reactivity during fear of males and females.

Parameter	Male mean (sd)	Female mean (sd)	Source	Df	F	Sig.
**Fear HR**	83.19 (4.658)	82.79 (3.441)	**Between groups**	1	.099	.755
			**Within groups**	44		
			**Total**	45		
**Fear RMSSD**	58.07 (9.77)	54.26 (7.49)	**Between groups**	1	2.041	.160
			**Within groups**	44		
			**Total**	45		
**Fear Ti**	1.56 (0.651)	1.39 (0.502)	**Between groups**	1	.870	.356
			**Within groups**	41		
			**Total**	42		
**Fear Te**	1.96 (0.841)	1.83 (0.514)	**Between groups**	1	.321	.574
			**Within groups**	41		
			**Total**	42		
**Fear Vt**	0.48 (0.51)	0.56 (0.616)	**Between groups**	1	.193	.663
			**Within groups**	41		
			**Total**	42		
**Fear Ttot**	3.48 (0.963)	3.17 (0.514)	**Between groups**	1	1.575	.217
			**Within groups**	41		
			**Total**	42		
**Δ Fear HR**	7.46 (14.258)	4.06 (11.415)	**Between groups**	1	.691	.411
			**Within groups**	40		
			**Total**	41		
**Δ Fear RMSSD**	1.92 (15.217)	-6.72 (10.098)	**Between groups**	1	4.350	.043*
			**Within groups**	40		
			**Total**	41		
**Δ Fear Ti**	0.186 (0.77)	0.246 (0.29)	**Between groups**	1	.089	.767
			**Within groups**	36		
			**Total**	37		
**Δ Fear Te**	0.428 (0.62)	0.28 (0.514)	**Between groups**	1	.611	.439
			**Within groups**	36		
			**Total**	37		
**Δ Fear Vt**	-0.062 (0.3)	0.041 (0.325)	**Between groups**	1	1.024	.318
			**Within groups**	36		
			**Total**	37		
**Δ Fear Ttot**	0.572 (0.94)	0.526 (0.65)	**Between groups**	1	.030	.864
			**Within groups**	36		
			**Total**	37		

sd: standard deviation; Df: degree of freedom; Sig: significance; HR: Heart rate; RMSSD: Root mean square of successive differences; Ti: Inspiration time; Te: Expiration time; Vt: Tidal volume; Ttot: Total breath time; Δ: Reactivity parameters.

*p<0.05

**p<0.01.

**Table 9:** ANOVA for emotion feedback form rating for all emotions by males and females.

Parameter	Male mean (sd)	Female mean (sd)	Source	Df	F	Sig.
**Feedback joy**	3.32 (1.056)	3.95 (0.89)	**Between groups**	1	5.037	.029*
			**Within groups**	48		
			**Total**	49		
**Feedback disgust**	3.39 (1.315)	4.05 (1.395)	**Between groups**	1	2.771	.103
			**Within groups**	46		
			**Total**	47		
**Feedback sad**	3.48 (1.252)	3.59 (1.098)	**Between groups**	1	.103	.749
			**Within groups**	47		
			**Total**	48		
**Feedback anger**	3.85 (1.23)	4 (0.976)	**Between groups**	1	.210	.648
			**Within groups**	47		
			**Total**	48		
**Feedback fear**	3.19 (1.039)	3.8 (1.152)	**Between groups**	1	3.668	.062
			**Within groups**	45		
			**Total**	46		

sd: standard deviation; Df: degree of freedom; Sig: significance

*p<0.05

**p<0.01.

The Bonferroni method was used to counteract the problem of multiple comparisons. Since there are total 71 variables, for the difference to be significant, the p value should be less than 0.05 / 71 i.e., 0.0007. Thus, no significant gender differences were found for any of the variables.

### Association between EI, autonomic response and reactivity

Bivariate correlations were used to assess the relationship between, cardiac response, cardiac reactivity, respiratory response and respiratory reactivity, with Emotional intelligence and gender which are given below.

#### Cardiac response and EI

**Fig 4:**
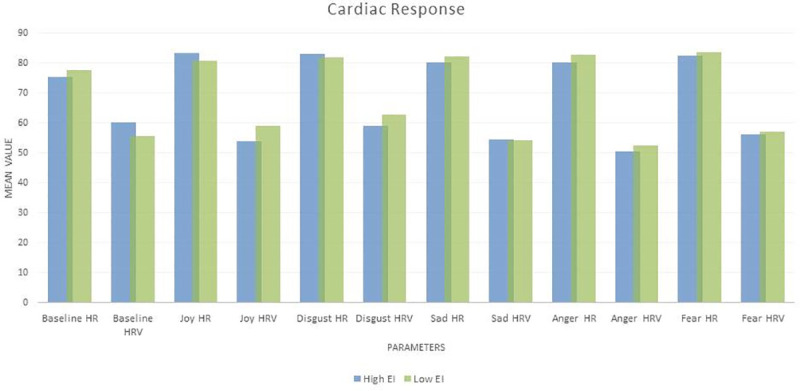
Mean cardiac response of high and low EI individuals.

The total EI score and all its sub- factors was associated with Baseline RMSSD. The total EI score along with the sub factors ‘perception of emotion’ and ‘managing own emotions’ showed a significant association with Disgust HR as well. Fig 4 below shows mean cardiac responses of high and low EI individuals (the EI scores were divided into 2 groups at the median–high and low EI) and Table 10 shows the Pearson correlations between them.

**Table 10:** Pearson correlation between cardiac response parameters and emotional intelligence scores.

	Total EI score	Perception of emotion	Managing Own emotions	Managing other’s emotions	Utilization of emotions
**Gender**	-0.019	-0.155	0.026	0.011	0.137
**Baseline HR**	-0.144	-0.18	-0.065	-0.239	0.081
**Baseline RMSSD**	.469**	.414**	.328*	.370*	.300*
**Joy HR**	0.045	0.103	0.017	-0.005	-0.013
**Joy RMSSD**	-0.22	-0.178	-0.255	0.002	-0.216
**Disgust HR**	.303*	.391**	.306*	-0.024	0.133
**Disgust RMSSD**	0.092	0.128	-0.02	0.089	0.085
**Sad HR**	0.132	0.193	-0.002	0.264	-0.083
**Sad RMSSD**	0.152	0.253	0.101	0.079	-0.053
**Anger HR**	-0.066	0.062	-0.179	0.035	-0.148
**Anger RMSSD**	-0.208	-0.082	-0.207	-0.171	-0.225
**Fear HR**	-0.053	-0.016	-0.051	0.069	-0.184
**Fear RMSSD**	0.238	0.223	0.239	0.023	0.217

EI: Emotional intelligence; HR: Heart Rate; RMSSD: Root Mean Square of Successive Differences.

*p<0.05

**p<0.01.

#### Cardiac reactivity and EI

**Fig 5:**
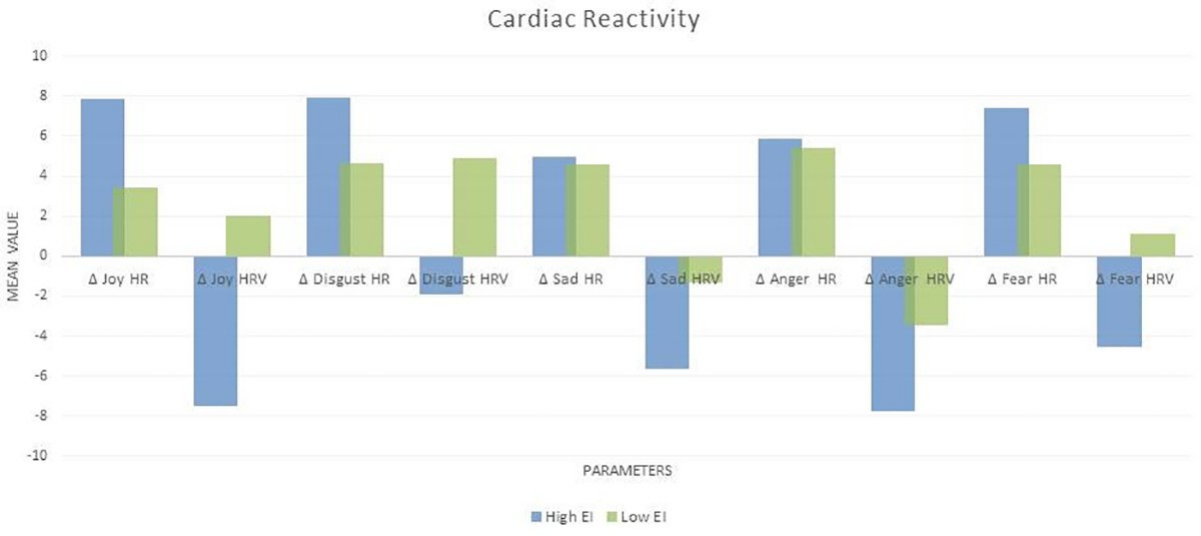
Mean cardiac reactivity of high and low EI individuals.

The total EI score was associated with Neutral RMSSD, Δ Disgust HR, Δ SAD RMSSD and Δ ANGER RMSSD. Of interest, EI score was not associated with cardiac response during joy. Sub-factors of EI–Perception of emotion, managing own emotions, managing other’s emotions, and utilization of emotions- also showed various significant correlations. All the sub factors were associated with Baseline RMSSD among others. Perception of emotion was associated with Δ Disgust HR, and Anger RMSSD. Managing other’s emotions showed association with Δ Disgust HR, Δ Sad RMSSD. Utilization of emotion was associated with Δ Joy RMSSD, and Δ Anger RMSSD. Surprisingly ‘Managing own emotions’ branch did not show any correlation with cardiac autonomic reactivity, nor did cardiac autonomic reactivity during fear show any correlation with EI scores. High EI individuals portrayed cardiac vagal withdrawal (RMSSD) during all 5 emotion elicitations while low EI individuals portrayed cardiac vagal activation during joy disgust and fear. Fig 5 below shows mean cardiac reactivity of high and low EI individuals and Table 11 shows the Pearson correlations between them.

**Table 11:** Pearson correlations between cardiac reactivity parameters and emotional intelligence scores.

	Total EI score	Perception of emotion	Managing Own emotions	Managing other’s emotions	Utilization of emotions
**Gender**	-0.019	-0.155	0.026	0.011	0.137
**Δ Joy HR**	0.13	0.185	0.049	0.203	-0.085
**Δ Joy RMSSD**	-.537**	-.498**	-.432**	-0.292	-.365*
**Δ Disgust HR**	0.211	0.268	0.135	0.233	-0.062
**Δ Disgust RMSSD**	-.343*	-0.27	-0.287	-0.27	-0.207
**Δ Sad HR**	0.172	0.222	0.061	.298*	-0.099
**Δ Sad RMSSD**	-.328*	-0.215	-0.233	-0.285	-.305*
**Δ Anger HR**	0.128	0.237	-0.01	0.27	-0.143
**Δ Anger RMSSD**	-.420**	-0.281	-.376*	-0.291	-.382*
**Δ Fear HR**	0.081	0.088	0.01	0.211	-0.063
**Δ Fear RMSSD**	-0.212	-0.182	-0.084	-0.254	-0.148

EI: Emotional intelligence; HR: Heart Rate; RMSSD: Root Mean Square of Successive Differences; Δ: Reactivity parameters.

*p<0.05

**p<0.01.

#### Respiratory response and EI

**Fig 6:**
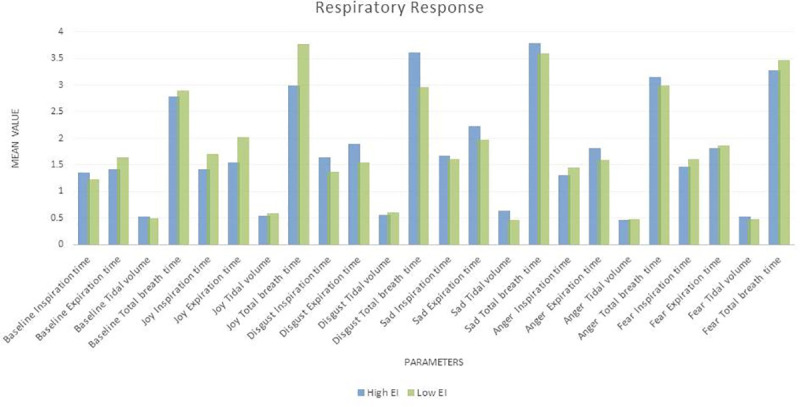
Mean respiratory response of high and low EI individuals.

The tidal volume during sadness was associated with Total EI and sub factors ‘perception of emotion’ and ‘managing other’s emotions’. Perception of emotion was also associated with tidal volume at baseline. Finally ‘utilization of emotion’ showed a significant correlation with tidal volume during both anger and fear. Fig 6 below shows mean respiratory response of high and low EI individuals and Table 12 shows the Pearson correlations between them.

**Table 12:** Pearson correlations between cardiac reactivity parameters and emotional intelligence scores.

	Total EI score	Perception of emotion	Managing own emotions	Managing other’s emotions	Utilization of emotion
**Gender**	-0.019	-0.155	0.026	0.011	0.137
**Baseline Ti**	0.226	0.169	0.265	0.061	0.188
**Baseline Te**	0.175	0.262	0.018	0.058	0.180
**Baseline Vt**	0.256	0.317*	0.134	0.035	0.272
**Baseline Ttot**	0.296	0.274	0.229	0.104	0.296
**Joy Ti**	-0.15	-0.253	-0.029	0.041	-0.169
**Joy Te**	0.111	0.136	0.063	0.034	0.084
**Joy Vt**	-0.003	0.059	-0.131	0.048	0.016
**Joy Ttot**	0.014	-0.008	0.012	0.087	-0.039
**Disgust Ti**	0.072	-0.024	0.179	-0.063	0.147
**Disgust Te**	0.158	0.074	0.288	-0.047	0.149
**Disgust Vt**	-0.123	0.011	-0.149	-0.063	-0.236
**Disgust Ttot**	0.117	-0.049	0.285	-0.092	0.252
**Sad Ti**	0.015	0.061	0.089	-0.147	-0.013
**Sad Te**	0.067	0.062	0.123	-0.017	0.006
**Sad Vt**	0.371*	0.332*	0.288	0.318*	0.164
**Sad Ttot**	0.057	0.011	0.159	-0.071	0.061
**Anger Ti**	-0.004	-0.131	0.075	-0.035	0.123
**Anger Te**	-0.025	-0.057	-0.058	-0.116	0.187
**Anger Vt**	-0.159	-0.128	-0.028	0.023	-0.396**
**Anger Ttot**	0.142	0.027	0.211	0.001	0.221
**Fear Ti**	0.059	-0.003	-0.062	0.089	0.223
**Fear Te**	0.039	0.135	-0.008	-0.064	0.021
**Fear Vt**	0.283	0.124	0.257	0.234	0.302*
**Fear Ttot**	-0.037	-0.034	-0.123	-0.012	0.088

EI: Emotional intelligence; Ti: Inspiration time; Te: Expiration time; Vt: Tidal volume; Ttot: Total breath time.

*p<0.05

**p<0.01.

#### Respiratory reactivity and EI

**Fig 7:**
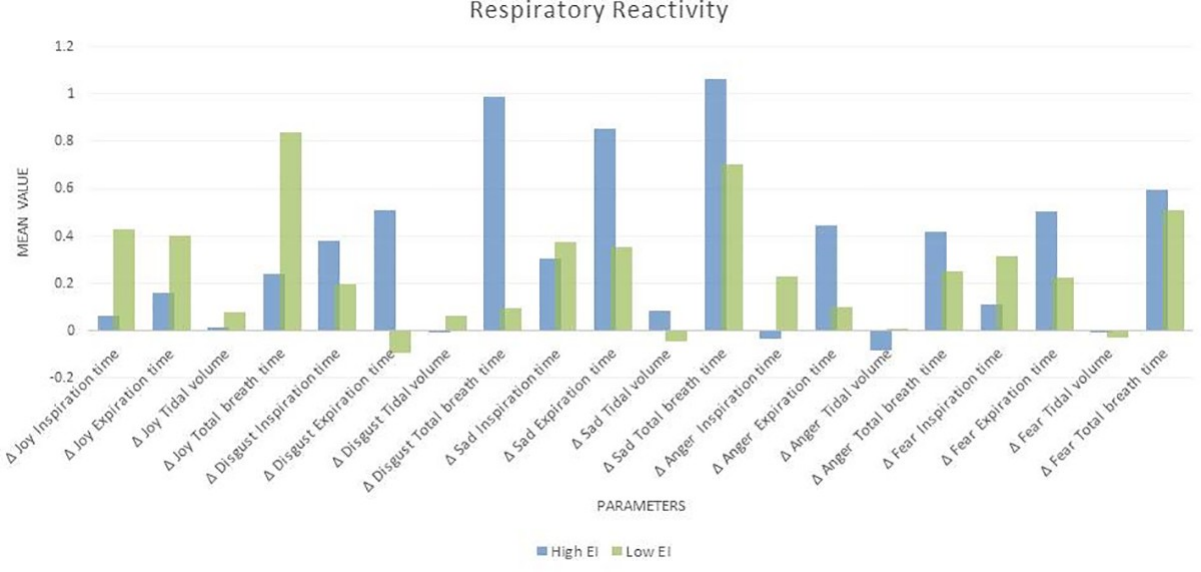
Mean respiratory reactivity of high and low EI individuals.

The total EI score and ‘utilization of emotion’ showed association with Δ Anger Tidal volume, the latter also showed association with Δ Disgust Tidal Volume. Perception of emotion was correlated with Δ Joy Inspiration time. Fig 7 below shows mean respiratory reactivity of high and low EI individuals and Table 13 shows the Pearson correlations between them. High and low EI individual showed significantly different activation and withdrawal of respiratory response during each emotion. Higher EI showed a lower respiratory reactivity to joy. During disgust high EI showed an increase in expiration time while low EI showed a decrease. In sadness, anger, and fear higher EI showed a much greater increase in expiration time (and as a result total breath time) from baseline and a lower increase in inspiration time from baseline.

**Table 13:** Pearson correlation between cardiac reactivity parameters and Emotional Intelligence scores.

	Total EI score	Perception of emotion	Managing own emotions	Managing other’s emotions	Utilization of emotion
**Gender**	-0.019	-0.155	0.026	0.011	0.137
**Δ Joy Ti**	-0.265	-0.339*	-0.128	-0.039	-0.273
**Δ Joy Te**	0.148	0.122	0.195	0.066	0.043
**Δ Joy Vt**	-0.081	-0.132	-0.008	0.079	-0.179
**Δ Joy Ttot**	-0.083	-0.091	-0.003	0.002	-0.179
**Δ Disgust Ti**	-0.031	-0.048	0.031	-0.057	-0.029
**Δ Disgust Te**	0.074	-0.053	0.227	-0.021	0.08
**Δ Disgust Vt**	-0.296	-0.189	-0.269	-0.049	-0.442**
**Δ Disgust Ttot**	-0.027	-0.125	0.141	-0.128	0.029
**Δ Sad Ti**	-0.115	-0.032	-0.034	-0.249	-0.096
**Δ Sad Te**	-0.019	-0.031	0.072	-0.045	-0.081
**Δ Sad Vt**	0.189	0.212	0.205	0.2	-0.099
**Δ Sad Ttot**	-0.139	-0.129	-0.026	-0.183	-0.111
**Δ Anger Ti**	-0.148	-0.146	-0.083	-0.136	-0.084
**Δ Anger Te**	-0.036	-0.059	-0.009	-0.036	0.007
**Δ Anger Vt**	-0.354*	-0.295	-0.157	-0.177	-0.509**
**Δ Anger Ttot**	-0.095	-0.121	-0.006	-0.116	-0.048
**Δ Fear Ti**	-0.128	-0.129	-0.212	-0.033	0.031
**Δ Fear Te**	-0.017	0.016	-0.026	-0.066	0.015
**Δ Fear Vt**	-0.064	-0.076	0.03	-0.004	-0.17
**Δ Fear Ttot**	-0.136	-0.1	-0.201	-0.081	-0.009

EI: Emotional intelligence; Ti: Inspiration time; Te: Expiration time; Vt: Tidal volume; Ttot: Total breath time; Δ: Reactivity parameters.

*p<0.05

**p<0.01.

### EI recognition results

**Fig 8:**
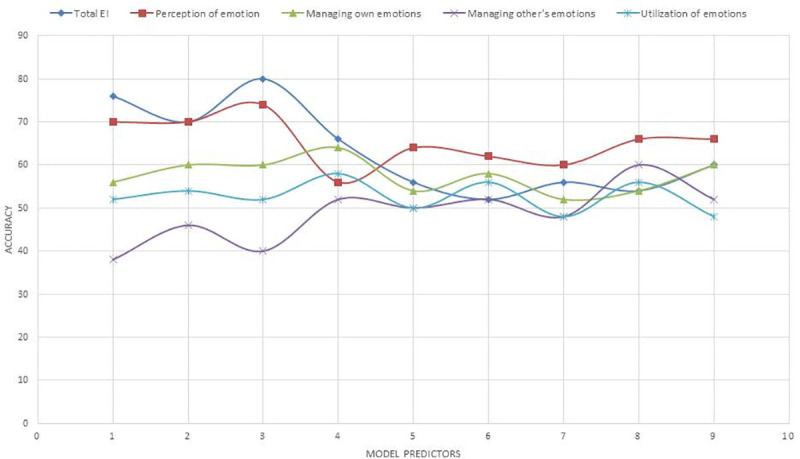
Model accuracy with linear SVM for different sets of predictors. 1: Cardiac response; 2: Cardiac reactivity; 3: Cardiac response and reactivity; 4: Respiratory response; 5: Respiratory reactivity; 6: Respiratory response and reactivity; 7: Autonomic (cardiac + breath) response; 8: Autonomic reactivity; 9: Autonomic response and reactivity.

**Fig 9:**
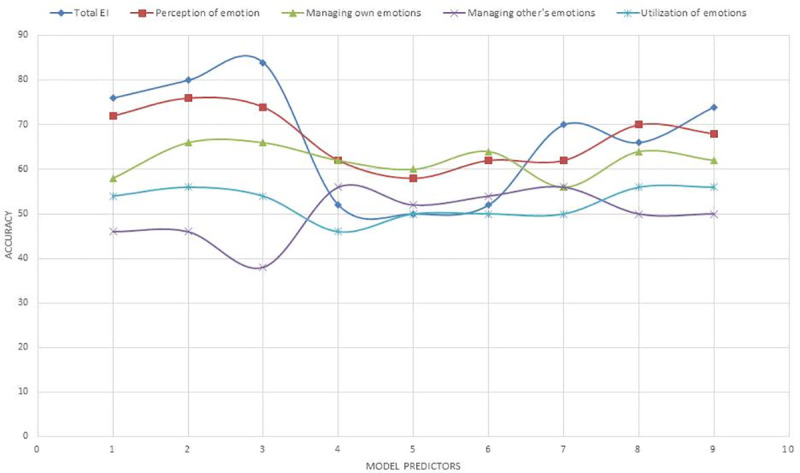
Model accuracy with linear SVM and PCA for different sets of predictors. 1: Cardiac response; 2: Cardiac reactivity; 3: Cardiac response and reactivity; 4: Respiratory response; 5: Respiratory reactivity; 6: Respiratory response and reactivity; 7: Autonomic (cardiac + breath) response; 8: Autonomic reactivity; 9: Autonomic response and reactivity.

For binary trait EI recognition, we first dichotomised the SSEIT scores based on the median. This resulted in an even distribution of high and low EI labels for the total EI score. We also checked whether the physiological response patterns to emotion could also predict each sub factor of EI, namely–Perception of Emotion, Managing own emotions, Managing other’s emotions, and Utilization of emotions which were also dichotomized based on their median. A 5 fold cross-validation scheme was used to compute the classification results and avoid over fitting. Different models were tested for predicting level of EI using different set of predictor variables to find out which set of autonomic predictors most efficiently predict level of EI. The set of predictor variables used for each model are: 1) Cardiac response, 2) Cardiac reactivity, 3) Cardiac response and reactivity, 4) Breath response, 5) Breath reactivity, 6) Breath response and reactivity, 7) Autonomic (cardiac + breath) response, 8) Autonomic reactivity, and 9) Autonomic response and reactivity. We also examined if applying PCA (with 99% variance) improved accuracy of the models. The model accuracies, before and after PCA, is shown in Figs 8 and 9 respectively. Table 14 presents the recognition results, with the best F1 scores achieved using each set of predictor variables denoted in bold.

**Table 14:** F1 scores for SVM.

Predictor variable sets	PCA	Total EI score	Perception of emotion	Managing own emotions	Managing other’s emotions	Utilization of emotion
**Cardiac response**	Before	0.75	0.75	0.59	0.46	0.58
	**After**	0.77	0.77	0.62	0.56	0.69
**Cardiac reactivity**	Before	0.68	0.75	0.62	0.57	0.65
	**After**	0.81	**0.80**	**0.70**	0.58	0.69
**Cardiac response and reactivity**	Before	0.77	0.77	0.64	0.46	0.64
	**After**	**0.85**	0.78	0.68	0.49	0.68
**Breath response**	Before	0.56	0.62	0.61	0.63	0.66
	**After**	0.61	0.64	0.60	0.67	0.62
**Breath reactivity**	Before	0.45	0.71	0.51	0.65	0.62
	**After**	0.51	0.66	0.62	0.64	0.67
**Breath response and reactivity**	Before	0.25	0.69	0.60	0.65	0.65
	**After**	0.56	0.64	0.68	0.66	0.65
**Autonomic response**	Before	0.42	0.67	0.56	0.62	0.57
	**After**	0.76	0.68	0.58	0.65	0.66
**Autonomic reactivity**	Before	0.41	0.70	0.55	**0.69**	0.66
	**After**	0.68	0.74	0.65	0.56	0.69
**Autonomic response and reactivity**	Before	0.47	0.73	0.58	0.61	0.55
	**After**	0.79	0.70	0.64	0.58	**0.71**

Cardiac response: HR and RMSSD during the emotion elicitation; Cardiac reactivity: Difference between HR and RMSSD at baseline and emotional state; Breath response: All breath parameters (Ti, Te, Vt, Ttot) during the emotion elicitation; Breath reactivity: Difference between breath parameters (Ti, Te, Vt, Ttot) at baseline and emotional state; Autonomic: both cardiac and respiratory parameters.

From the recognition results we can provide following conclusions: Trait Emotional intelligence leads to changes in the physiological response to different emotions measured as autonomic response and reactivity to film clips. For total EI level recognition a better than chance recognition F1 score (>0.5) is achieved with all the 9 models after PCA. For the EI sub factor level recognition also, a better than chance recognition F1 score (>0.5) is achieved with all the 9 models after PCA except for prediction of ‘managing other’s emotions’ sub factor from ‘cardiac response and reactivity’.

Best F1 score for EI prediction is achieved from cardiac response and reactivity (0.85) with PCA. Considering the sub factors, highest F1 scores is achieved for ‘perception of emotion ability’ (0.80). For both total EI and EI sub factors prediction, highest Accuracy and F1 Score is achieved from cardiac parameters, except ‘utilization if emotion’ which was predicted best by autonomic (cardiac + breath) response and reactivity (F1 = 0.71).

## Discussion and conclusion

In the current study, we first explored the relationship between trait EI and autonomic emotional response patterns and then examined the accuracy of the latter in predicting level of EI using linear SVM. To the best of our knowledge, only one study has been conducted for predicting EI using physiological measures, by IIT Delhi and IBM research group. They used standardised multiple choice questions as ground truth measure of EI which was indicated using audio signal parameters (like initial response time, pause time etc.) while responding to interview questions, and Watson personality insights tool which is a service on the IBM cloud that predicts personality characteristics, needs and values through written text [88]. Despite various studies examining EI and its association with emotional response, no work has been done on modelling trait EI based on emotional behaviour. We also explored gender differences regarding EI scores, autonomic emotional response patterns and emotion feedback rating for 5 basic emotion elicitations–joy, disgust, sadness, anger, and fear. No significant gender differences were found for any of the variables. Earlier studies have reported varying results regarding the difference in EI in males and females, ranging from women having relatively higher EI than men [89], among creative professionals men having higher EI than women [89], and among nursing students women reportedly having higher EI than men [90]. Research has shown that low EI males have lower parasympathetic and higher sympathetic activity at rest and are thus more prone to developing hypertension [91]. Two of the trait EI factors ‘perception of emotions’ and ‘managing own emotions’ have also shown significant correlation with cardiovascular measures of emotional arousal during sadness and cheerfulness in females [92]. Higher EI has also been found to be associated with higher cardiac autonomic flexibility [93]. Earlier studies have also shown that gender differences in emotional experience vary with the type of emotion, which have been found particularly strongly for anger and fear as men and women differ in how intensely they experience and inhibit these emotions [94]. Even though the difference was not statistically significant, it was observed that the average feedback form rating for all the emotions was higher in females than males (Table 9). A recent study similarly reported that while watching emotion eliciting videos, men have more intense emotional experience (physiological arousal evoked by external stimuli) and women have higher emotional expressivity (external expression of subjective experience including self-report) [95]. Since emotional experience and expressivity belong to different reaction systems, the inconsistency between them is understandable and has been previously observed by various researchers [95–97].

The few studies done on the association between trait EI and physiological responses show inconsistent results, a recent work by Zysberg found no association between trait EI and the cardiac vagal regulation during self-induced sadness unlike what was observed in the meta-analysis recently done by Ainize et al. [98] Thus, it should be highlighted that there is a lack of research on the effects of TEI on biological variables, other than the few mentioned above, not much is known about the physiological correlates of trait EI [69]. After exploring gender differences, we invstigated the association between trait EI and autonomic response and reactivity to emotion eliciting videos. Emotional intelligence and emotion regulation are highly related frameworks. Emotional intelligent individuals are those who carefully review the context before deciding whether and how they should regulate their emotion, and are able to do so in a flexible adaptive manner [1]. High EI showed significant association with RMSSD at baseline indicating high emotion regulation capacity and autonomic flexibility, [40] which is in line with previous research as EI is associated with efficient handling of emotions personally and socially as low HRV at baseline is associated with difficulties in emotion regulation [39]. Maximum significant correlation were observed between Total EI score and all the physiological parameters followed by sub factor ‘perception of emotion’. Least correlation with the physiological parameters was observed with the sub factor ‘managing other’s emotions’ which may be due to lack of any measure pertaining specifically to social interaction. Participants with higher trait EI exhibited a cardiac vagal withdrawal indicated by decrease in RMSSD from baseline to emotional state. Consistent with previous studies, wherein higher EI individuals showed increased reactivity to emotion eliciting videos, [1] we found that high EI individuals had a greater increase in heart rate in response to all 5 emotions as compared to their low EI counterparts indicating higher emotional reactivity in high EI individuals. With respect to respiratory parameters, EI scores correlated mostly with Tidal volume. The significantly greater increase in expiration time by higher EI individuals during negative emotions (disgust, sadness, anger, and fear) shows the individual’s attempt to relax as increasing expiration time activates the parasympathetic nervous system putting the body in a state of rest and digest [99]. This result explains the increased subjective resistance to negative emotions and lower negative state affectivity of higher EI individuals [58, 66]. Emotion elicitation results in a modification in ANS activity due to emotional arousal and reactivity and a significant difference was observed in emotional reactivity of high and low EI individuals which explains how high EI individuals fundamentally differ from low EI individuals in how they handle various emotions. This is in line with previous research wherein an EEG study showed association between trait EI and frontal asymmetries indicating difference in emotional dispositions of high and low EI individuals [100]. Our results show that higher EI individuals experience all emotions to the fullest and also attempt to regulate them in order to maintain a balance. They do not suppress emotional experinece neither do they constantly regulate negative emotions. They are sensitive to affective cues and leave room for emotions to emerge [1].

For EI prediction an SVM machine learning algorithm was used along with PCA with 99% variance. For EI level prediction we achieved an F1 score of 0.85 and accuracy of 84% with cardiac response and reactivity. A better than chance F1 score (<0.5) and accuracy were also achieved for prediction of all the sub factors of EI. Highest accuracy and F1 scores for prediction of sub factors ‘perception of emotion’ (F1 = 0.80, Accuracy = 76%), and ‘managing own emotions’ (F1 = 0.70, Accuracy = 66%) was obtained using only cardiac reactivity as predictor. ‘Managing other’s emotions’ (before PCA, F1 = 0.69, Accuracy = 60%) and ‘Utilization of emotion’ (F1 = 0.71, Accuracy = 56%) were best predicted by autonomic reactivity, and autonomic response and reactivity respectively.

This work makes the following research contributions: (i) We studied gender differences with respect to EI score, physiological response and reactivity to various emotions and the emotional feedback form rating; (ii) We examined the association between trait EI and autonomic response and reactivity to five emotions–joy, disgust, sadness, anger and fear–which showed how an individual’s trait EI can plays a role in the way they physiologically respond to emotion elicitation; (iii) Emotional Intelligence has traditionally been assessed via the questionnaires, or by maximal performance tests. Differently we recognize the level of trait EI by examining psychophysiological emotional response patterns. It is the first step towards creating a physiological indicator of trait EI. The only other work in this domain is by Agarwal P. et al. [88] which used audio parameters along with a personality test to predict EI with an accuracy of 67%; and (iv) we use *non-contact based*, *off-the-shelf* sensors for physiological recordings and also the prediction model only uses physiological measures. This enhances the ecological validity of our work, and affirms its utility and promise for commercial applications. We have uploaded the dataset for future use.

## Limitations and future directions

This study has various limitations that cannot be ignored. Firstly, as mentioned earlier a measure pertaining to social interaction needs to be included, to make the prediction more accurate. Other Trait EI measures should be taken into consideration for creating a more detailed model. This study was conducted on students of IIT Kharagpur, a more diverse sample size both with respect to age and location should be studied to observe any cultural or age-related differences. Overall further research needs to be conducted to gain better understanding of EI, emotions, affect, physiological response to emotions, and their association with one another. Lastly for cardiac measures only RMSSD was considered, other time domain and frequency domain parameters should be considered in future studies.

In future work, the model accuracy can be improved by adding parameters that measure specific facets of EI such as sociability, empathy etc. of an individual e.g., eye tracker data can be used to observe whether the individuals focus on faces or on inanimate objects on a given screen which has been shown to indicate social anxiety in an individual [101]. Also emotional repair after the emotional video ends could also be measured for a complete assessment of emotional response patterns of an individual. Several other machine learning models could also be applied for improving EI prediction performance. Future studies can also explore why emotional experience and expressivity differ from each other. Exploring the connection of EI with emotional experience, expressivity, and emotion regulation strategies will aid in developing a more accurate and reliable system for EI prediction.

## Supporting information

S1 File Dataset. (XLSX)
